# Frailty Transition Among Older Adults Living With HIV in Thailand: A 5‐Year Longitudinal Study

**DOI:** 10.1002/jia2.70099

**Published:** 2026-04-01

**Authors:** Tanakorn Apornpong, Win Min Han, Akarin Hiransuthikul, Hay Mar Su Lwin, Napon Hiranburana, Sasiwimol Ubolyam, Stephen J. Kerr, Thira Woratanarat, Anchalee Avihingsanon, HIV‐NAT 006 and 207 study team

**Affiliations:** ^1^ HIVNAT, Thai Red Cross AIDS and Infectious Diseases Research Centre Bangkok Thailand; ^2^ Faculty of Medicine, Department of Preventive and Social Medicine Chulalongkorn University Bangkok Thailand; ^3^ Center of Excellence in Tuberculosis, PulmonaryDepartment of Medicine Chulalongkorn University Bangkok Thailand; ^4^ The Kirby Institute University of New South Wales Sydney New South Wales Australia; ^5^ Faculty of Medicine, Research Affairs Chulalongkorn University Bangkok Thailand

**Keywords:** elderly PWH, frailty phenotypes, frailty transition, frailty, multimorbidity, pre‐frailty, PWH, vitamin D

## Abstract

**Introduction:**

Frailty is highly prevalent among older people with HIV (PWH), driven by multimorbidity and HIV‐associated accelerated ageing. We investigated frailty transitions and associated factors over a 5‐year follow‐up period in an ageing cohort of PWH in Thailand.

**Methods:**

We conducted a prospective cohort study among PWH aged ≥50 years in Bangkok, Thailand, between May 2015 and June 2024. Frailty phenotypes were assessed at baseline and at 5 years of follow‐up using the Fried frailty phenotype, including unintentional weight loss, low physical activity, exhaustion, weak grip strength and slow walking speed. Multinomial logistic regression was performed to identify factors associated with frailty progression and reversibility over follow‐up.

**Results:**

Among 324 participants enrolled (63% male; median age of 54 [IQR, 52−59] years), 158 (49%) were robust, 153 (47%) were pre‐frailty and 13 (4%) were frailty at baseline. Over 5 years, 111 participants (34%) experienced frailty worsening, 158 (49%) remained stable and 55 (17%) demonstrated frailty reversal. Among 158 PWH who were robust at baseline, 75 (47%) remained robust, 57 (36%) transitioned to pre‐frailty and 26 (16%) progressed to frailty. Notably, among those frail at baseline (*N* = 13), 65% improved to pre‐frailty or robustness. Low physical activity was the most common frailty component at baseline, while weak grip strength was the most predominant frailty phenotype at year 5. In multivariable analysis, multimorbidity (adjusted odds ratio [aOR] 3.09, 95% confidence interval [CI]: 1.42−6.72, *p* = 0.004), antiretroviral therapy (ART) duration>20 years (aOR 1.82, 95% CI: 1.08−3.06, *p* = 0.025) and baseline vitamin D deficiency (aOR 1.85, 95% CI: 1.10−3.10, *p* = 0.019) were independently associated with increased frailty over the 5‐year follow‐up. Conversely, multimorbidity (aOR 0.44, 95% CI: 0.44 [0.23−0.84], *p* = 0.013) and CD4 counts< 500 cells/mm^3^ (aOR 0.25, 95% CI: 0.10−0.62, *p* = 0.003) were associated with a lower likelihood of frailty reversal.

**Conclusions:**

Frailty among PWH aged ≥50 years in Thailand was common and highly dynamic, with over half of frail participants improving over 5 years. Multimorbidity, prolonged ART exposure and vitamin D deficiency were key predictors of frailty progression, whereas CD4< 500 cells/mm^3^ and multimorbidity reduced the likelihood of frailty reversal. These findings highlight frailty as modifiable and support integrating routine frailty screening and geriatric‐informed care into long‐term HIV services.

**Clinical Trial Registration:**

NCT00411983

**Abbreviations**
ART: antiretroviral therapy
ASCVD: atherosclerotic cardiovascular disease
ASMI: appendicular skeletal muscle mass index
AWGS: Asian Working Group for Sarcopenia
BMD: bone mineral density
BMI: body mass index
CI: confidence interval
CKD: chronic kidney disease
CVD: cardiovascular disease
DEXA: dual‐energy X‐ray absorptiometry
DM: diabetes mellitus
FFP: Fried frailty phenotype
GEE: generalized estimating equations
HBV: hepatitis B
HDL: high‐density lipoprotein
HIV‐NAT: HIV Netherlands Australia Thailand research collaboration
hs‐CRP: high sensitivity C‐reactive protein
HT: hypertension
IL‐6: interleukin‐6
INSTIs: integrase strand transfer inhibitors
IQR: interquartile range
kg: kilogram
kg/m^2^
: kilograms per square metre
m/s: metres per second
MetS: metabolic syndrome
mg/dL: milligrams per decilitre
MNA: Mini Nutritional Assessment
MoCA: Montreal Cognitive Assessment
NCI: neurocognitive impairment
ng/mL: nanograms per millilitre
NNRTI: non‐nucleoside reverse transcriptase inhibitor
OR: odds ratio
PHQ‐9: Patient Health Questionnaire‐9
PWH: people with HIV
SD: standard deviation
TDI: Thai Depression Inventory

## 1 Introduction

Globally, the population of older people with HIV (PWH) has increased substantially due to the effectiveness and widespread use of combination antiretroviral therapy, resulting in life expectancies that are now similar to those of people without HIV [1]. However, ageing with HIV is associated with increased risk for chronic diseases driven by both age‐related biological processes and persistent inflammation. Chronic oxidative stress [2] and ongoing immune activation [3] contribute to earlier onset and higher rates of comorbidities among PWH, often occurring 5–10 years earlier than in the general population [4]. Consequently, older PWH experience a higher burden of multimorbidity, where two or more chronic age‐related diseases can interact and exacerbate one another, such as diabetes mellitus (DM) [5], cardiovascular diseases (CVDs) [6], cancer [7] and frailty [8]. As a result of this, both multimorbidity and increased risk of associated polypharmacy substantially complicate clinical management of older PWH [9].

Frailty is a geriatric syndrome characterized by reduced physiological reserve and increases vulnerability to stressors, including social and environmental determinants [10]. Among PWH, frailty is associated with a markedly higher risk of adverse health outcomes, including falls, hospitalization, disability, depression, reduced quality of life, cognitive impairment, and increased morbidity and mortality [11, 12].

A recent systematic review and meta‐analysis reported a frailty prevalence of 10.9% and a pre‐frailty prevalence of 47.2% in PWH aged >50 years [13], with incidence rates ranging from 7.0% to 51.4% depending on the assessment method and the length of follow‐up [14]. Although frailty causes a significant morbidity and mortality burden, it is dynamic in nature, and individuals may transition between different frailty states over time [15], including reversal to pre‐frail or, infrequently, to non‐frail status. Routine frailty assessments are, therefore, essential to monitor longitudinal changes, identify individuals at risk and better understand factors associated with frailty progression or improvement.

Despite growing recognition of frailty in PWH [10], evidence on frailty transitions and their determinants, especially in resource‐constrained settings, remain limited. This study investigated bidirectional frailty transitions, including both progression and reversibility, and their associated factors over a 5‐year follow‐up period in a cohort of older PWH in Thailand.

## 2 Methods

### 2.1 Study Population and Design

This prospective longitudinal study included PWH aged ≥50 years receiving routine HIV care at HIVNAT, Thai Red Cross AIDS and Infectious Diseases Research Centre, Bangkok, Thailand. Frailty assessments were performed at baseline (2015−2017), and repeated at a 5‐year follow‐up visit. All participants who completed baseline frailty assessments were included in the analysis.

### 2.2 Study Outcomes

Frailty was assessed using a modified Fried frailty phenotype (FFP), which includes adaptations to the weight loss and physical activity components. The FFP consists of five criteria: unintentional weight loss, exhaustion, weak grip strength, low physical activity and slow walking speed. Unintentional weight loss was defined as a documented loss of ≥4.5 kilogram (kg) or ≥5% of body weight over 12 months, or ≥2.5% over 6 months for participants with <12 months of follow‐up [16]. Exhaustion was assessed using two items from the Center for Epidemiologic Studies Depression (CES‐D) scale referring to fatigue experienced for 1–2 days in the past week. Grip strength was measured using a hand‐held dynamometer and adjusted for body mass index (BMI) and sex. Low physical activity was determined using self‐reported limitations in strenuous activities (e.g. running or lifting heavy objects) based on the physical functioning domain of the SF‐36 Health Survey [17, 18]. Walking speed was determined using the best of two 4‐metre walk trials and adjusted for sex and height. Established cutoff points were used for weak grip strength and slow walking speed [19].

Each frailty component was scored as 1 (abnormal) or 0 (normal), yielding a total score categorized as normal or robust (0), pre‐frail (1−2) or frail (≥3).

The primary outcomes were change in frailty status from baseline to 5‐year visit as follows: (1) worsening, defined as a progression from robust to pre‐frail or frail, or from pre‐frail to frail; and (2) reversibility, defined as an improvement from frail to pre‐frail or robust, or from pre‐frail to robust.

### 2.3 Study Measurements

Demographics, clinical characteristics and behavioural data were extracted from electronic medical records, including sex, age, smoking status, alcohol use, fracture history and comorbidities (i.e. DM, hypertension [HT], chronic kidney disease [CKD], hepatitis B virus [HBV] and hepatitis C virus [HCV] and multimorbidity) at baseline and year 5 visit. Body composition was evaluated using BMI and waist‐to‐hip ratio. Metabolic syndrome (MetS) was defined by the presence of ≥3 of the following: waist circumference ≥90 centimetres (cm) (men) or ≥80 cm (women); fasting blood glucose ≥100 milligrams per decilitre (mg/dL) or DM diagnosis; blood pressure ≥130/85 mmHg or use of antihypertensive drug; triglycerides ≥150 mg/dL or lipid‐lowering therapy; and high‐density lipoprotein (HDL) cholesterol <40 mg/dL (men) or <50 mg/dL (women).

Bone mineral density (BMD) was assessed by using dual‐energy X‐ray absorptiometry (DEXA) scan and classified by T‐scores as normal (> −1), osteopenia (−1 to −2.5), or osteoporosis (< −2.5). Sarcopenia was assessed using appendicular skeletal muscle mass index (ASMI) measured by a multi‐channel bioelectrical impedance analysis device (BC‐148). The definition of sarcopenia as per Asian Working Group for Sarcopenia (AWGS) criteria were: ASMI <7.0 kilograms per square metre (kg/m^2^) (men) and <5.7 kg/m^2^ (women), handgrip strength <28 kg (men) or <18 kg (women), or walking speed <1.0 metres per second (m/s). Inflammatory biomarkers (high‐sensitivity C‐reactive protein [hs‐CRP], interleukin‐6 [IL‐6]) and vitamin D levels were measured at baseline only. Vitamin D deficiency was defined as a serum 25(OH)D levels <20 nanograms per millilitre (ng/mL), insufficiency as 20–30 ng/mL and normal levels as >30 ng/mL.

HIV‐related parameters included antiretroviral therapy (ART) regimens, nadir and current CD4 cell count, CD4/CD8 ratio, time since HIV acquisition and ART duration. Ten‐year atherosclerotic cardiovascular disease (ASCVD) risk was estimated using the pooled cohort equation [20], and categorized as low risk (<5%) or borderline to high risk (≥5%).

Neurocognitive function, nutritional status and depression were assessed at baseline and at the 5‐year visit using the Thai Montreal Cognitive Assessment (MoCA‐T), Mini Nutritional Assessment (MNA), Thai Depression Inventory (TDI) and Patient Health Questionnaire‐9 (PHQ‐9). Neurocognitive impairment (NCI) was defined as a MoCA‐T score <25, with a 1‐point adjustment for participants with ≤6 years of education [21, 22]. Abnormal nutritional status was defined as an MNA score <23.5, indicating at risk for malnutrition or malnourishment. Depression was evaluated using the TDI at baseline and PHQ‐9 at follow‐up. TDI scores were categorized as no depression (0–20) or mild to severe depression (21–41). The PHQ‐9 was categorized as none to minimal (0–4) or mild to severe depression (5–27).

Multimorbidity was defined as the presence of ≥3 chronic comorbid conditions, irrespective of the sequence of onset or disease severity [4]. Comorbid conditions for multimorbidity were identified based on medical diagnoses or treatments: NCI, CVD, pulmonary conditions, HT, DM, obesity (BMI >30 kg/m^2^), MetS, dyslipidaemia, cancer, musculoskeletal, CKD, HBV and mental health conditions.

### 2.4 Ethical Consideration

This study received approval from the Ethics Committee of the Faculty of Medicine, Chulalongkorn University (approval # 0883/67). Written informed consent was obtained from all participants.

### 2.5 Statistical Analysis

Descriptive statistics were presented according to the type of variables among the frailty status as robust, prefrail and frail groups. Continuous variables were presented as the median with interquartile range (IQR); categorical variables were summarized as frequencies and percentages. Comparisons between groups were conducted using Kruskal−Wallis tests for continuous variables, and Fisher's exact tests for categorical variables, as appropriate. Frailty prevalence was assessed at baseline and the 5‐year visit. The cumulative incidence of frailty was calculated among participants who were robust or pre‐frail at baseline.

Generalized estimating equations (GEE) with binomial family, logit link and exchangeable correlation matrix were used to identify factors associated with frailty and frailty/pre‐frailty at baseline and at the 5‐year follow‐up. We then used multinomial logistic regression to evaluate baseline factors associated with frailty transition >5 years, categorized as worsening, stable or reversal, with stable frailty status as the reference group. Effect estimates are presented as odds ratios (ORs) with 95% confidence intervals (CIs).

In both analytical models, participants who were lost to follow‐up or who died after baseline were imputed as frail at the 5‐year visit. No imputation was performed for individual frailty components or other clinical parameters. The following predictor covariates were included in the models: age, sex, BMI, alcohol and smoking consumption, HT, DM, HBV, HCV, CKD, multimorbidity, ASCVD scores, NCI, nutrition status, BMD, fracture history, sarcopenia, ART regimen, current CD4, inflammatory biomarkers (IL‐6, hs‐CRP) and vitamin D concentrations. For GEE analysis, predictor covariates were modelled at baseline and follow‐up, with last observation carried forward for participants who died or were lost. In multinomial models, only predictor covariates at the baseline visit were modelled. In both GEE and multinomial models, multivariable models were adjusted for factors significant in univariable models at ≤0.2; multivariable models were also adjusted for age and sex, given their known association with frailty [23]. To minimize multicollinearity, multimorbidity was selected for the multivariable model instead of individual comorbidities. Multicollinearity for model covariates were checked using variance inflation factors, and *p*‐values <0.05 were considered statistically significant. All analyses were performed using Stata version 18.5 (StataCorp LLC, College Station, TX, USA).

## 3 Results

### 3.1 Participants’ Characteristics at Baseline

Of 358 PWH enrolled, 324 (90.5%) completed frailty assessment at baseline and were included in the analysis. Participant characteristics stratified by frailty status are summarized in Table 1. The majority were male (63%), with a median age of 54 (IQR: 52–59) years. At baseline, 43 (13.3%) were current smokers and 27 (8.3%) reported current alcohol consumption. The median duration of ART was 16 (13−19) years, with approximately half receiving non‐nucleoside reverse transcriptase inhibitor (NNRTI)‐based regimens. The median (IQR) number of comorbidities was 3 (2−4) diseases, and 75% met criteria for multimorbidity. The five most common comorbidities were dyslipidaemia (87%), NCI (74%), HT (44%), MetS (42%) and DM (18%).

**TABLE 1:** Demographic characteristics and baseline frailty status.

	Frailty status at baseline			
Characteristics	Robust (*n* = 158)	Pre‐frail (*n* = 153)	Frail (*n* = 13)	*p*‐value
**Age, year**	54 (52−57)	55 (52−60)	59 (54−63)	0.012
**Sex**				0.073
Male	107 (67.72)	92 (60.13)	5 (38.46)	
Female	51 (32.28)	61 (39.87)	8 (61.54)	
**Ever smoked**	58 (36.71)	54 (35.29)	3 (23.08)	0.662
**Ever consumed alcohol**	36 (22.78)	31 (20.26)	2 (15.38)	0.809
**BMI ≥25 kg/m^2^**	33 (20.89)	44 (28.76)	7 (53.85)	0.022
**Waist‐hip ratio**	0.93 (0.880.97)	0.92 (0.870.97)	0.91 (0.890.95)	0.885
**Metabolic syndrome**	63 (39.87)	66 (43.14)	9 (69.23)	0.126
**Hepatitis B**	23 (14.56)	17 (11.11)	2 (15.38)	0.607
**Hepatitis C**	15 (9.49)	14 (9.15)	1 (7.69)	1.000
**Diabetes mellitus**	26 (16.46)	26 (16.99)	3 (23.08)	0.782
**Hypertension**	57 (36.08)	71 (46.41)	8 (61.54)	0.062
**Chronic kidney disease**	10 (6.33)	14 (9.15)	5 (38.46)	0.004
**Number of comorbidities**	3 (2−4)	3 (2−4)	4 (3−6)	0.009
**Multimorbidity**	92 (58.23)	101 (66.01)	11 (84.62)	0.101
**Duration year on ART**	16 (11−19)	16 (14−19)	19 (16−20)	0.027
**CD4 cell count (cells/mm^3^)**	584 (443−782)	652 (513−814)	603 (528−898)	0.045
**HIV‐RNA ≤50 copies/mL**	153 (96.84)	149 (97.39)	13 (100)	1.000
**ART regimen**				0.067
NNRTI‐based	99 (62.66)	75 (49.02)	5 (38.46)	
PI‐based	41 (25.95)	59 (38.56)	6 (46.15)	
Others	18 (11.39)	19 (12.42)	2 (15.38)	
**Nutrition status**				0.488
Normal	133 (84.18)	122 (79.74)	10 (76.92)	
Abnormal	25 (15.82)	31 (20.26)	3 (23.08)	
**Depression**	7 (4.43)	12 (7.84)	1 (7.69)	0.353
**ASCVD risk score**	6.1 (3.3−10.6)	6.6 (2.7−14.2)	6.3 (3.7−11.8)	0.883
**Cognitive impairment**	89 (56.33)	94 (62.25)	10 (76.92)	0.269
**Number of medications including ART**	5 (4−6)	5 (4−6)	8 (5−9)	0.019
**Bone mineral density at hip**				0.578
Normal	104 (68.87)	96 (65.31)	9 (69.23)	
Osteopenia	44 (29.14)	47 (31.97)	3 (23.08)	
Osteoporosis	3 (1.99)	4 (2.72)	1 (7.69)	
**Fracture history**	4 (2.53)	6 (3.92)	1 (7.69)	0.36
**Sarcopenia (*n* = 282)** ^a^	9/138 (6.52)	8/134 (5.97)	0/10 (0.00)	1.000
**Interleukin‐6 (pg/mL)**	5.8 (3.8−7.4)	6.4 (4.5−9.7)	6.3 (5.3−11.8)	0.017
**hs‐CRP, (mg/L)**	1.1 (0.5−2.6)	1.2 (0.6−2.8)	1.7 (1−2.1)	0.623
**Vitamin D status (**25‐hydroxyvitamin D), **(ng/mL)**				0.969
Normal (>30)	16 (10.32)	19 (12.58)	1 (7.69)	
Insufficiency (20−30)	85 (54.84)	78 (51.66)	7 (53.85)	
Deficiency (<20)	54 (34.84)	54 (35.76)	5 (38.46)	
**TSH >10 mIU/L**	119 (75.32)	113 (73.86)	12 (92.31)	0.357

*Note*: Data are presented as median (interquartile range) for continuous variables and frequency (percentage) for categorical variables. Statistical significance was assessed using the Kruskal–Wallis test for continuous variables and the chi‐square or Fisher's exact test for categorical variables, as appropriate. Frailty status was defined using the Fried frailty phenotype at baseline.

Abbreviations: ART, antiretroviral therapy; ASCVD, atherosclerotic cardiovascular disease; BMI, body mass index; CRP, C‐reactive protein; hs‐CRP, high sensitivity CRP; NNRTI, non‐nucleoside reverse transcriptase inhibitor; PI, protease inhibitor; TSH, thyroid‐stimulating hormone.

^a^Sarcopenia was assessed in participants with available body composition data (*n* = 282).

### 3.2 Characteristics at 5‐Year Follow‐Up Visit

After 5 years of follow‐up, 272 participants (84%) were reassessed for frailty. Reasons for missing follow‐up assessments included death (31, 9.6%) and loss to follow‐up (21, 6.5%). Among those who died, 23 deaths were attributed to illness‐related causes, one to suicide and seven had unknown causes of death. Baseline frailty status among deceased participants was classified as robust in 13 (41.9%), pre‐frail in 14 (45.2%) and frail in 4 (12.9%). Median (IQR) time between the first and second assessments was 5.92 (5.60−6.84) years. Baseline characteristics of participants with and without follow‐up assessments are reported in Table S1.

After 5 years of follow‐up, only 35 participants (10.8%) remained on NNRTI‐based regimens, while 138 (42.6%) had switched to integrase strand transfer inhibitors (INSTIs)‐based regimens. Seventy‐five percent of participants had multimorbidity. The most common comorbidities were dyslipidaemia (84%), NCI (77%), MetS (52%), HT (51%) and DM (41%). Frail participants had a higher prevalence of past smoking, multimorbidity and abnormal nutritional status compared with pre‐frail and robust individuals (Table S2).

### 3.3 Prevalence and Incidence of Frailty

At baseline, 158 (48.8%) were robust, 153 (47.2%) were pre‐frail and 13 (4.0%) were frail. The most common abnormal frailty phenotype was low physical activity (33.3%), followed by unintentional weight loss (15.4%) and weak grip strength (14.5%).

**FIGURE 1:**
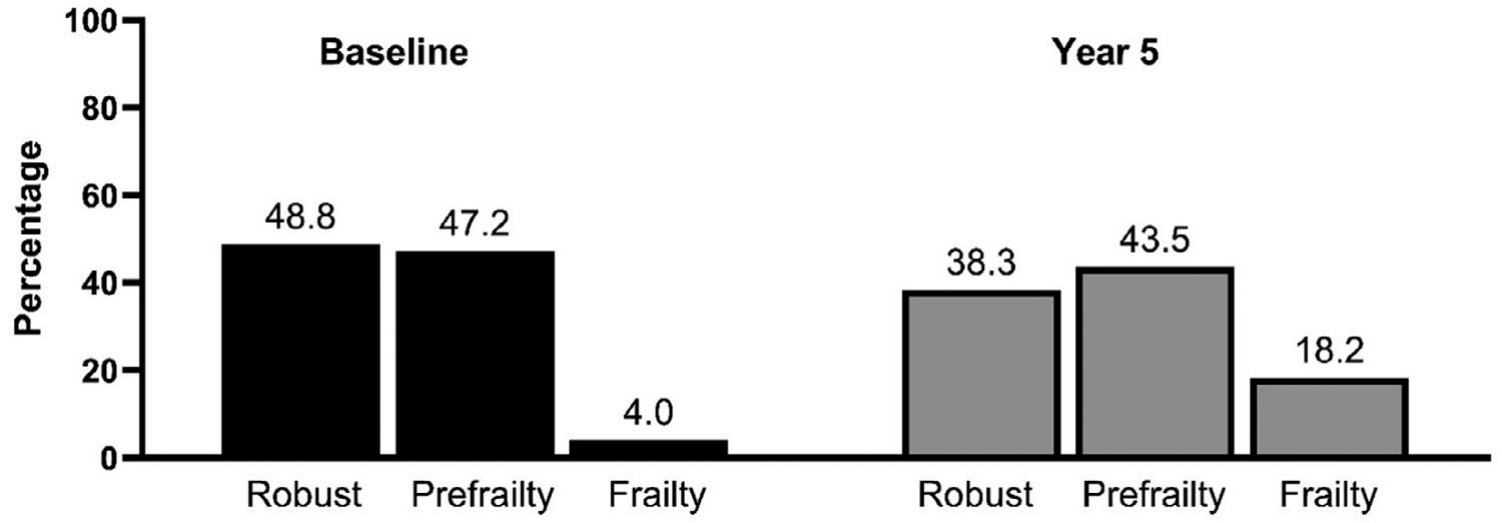
Prevalence of frailty at baseline and after 5 years of follow‐up.

**FIGURE 2:**
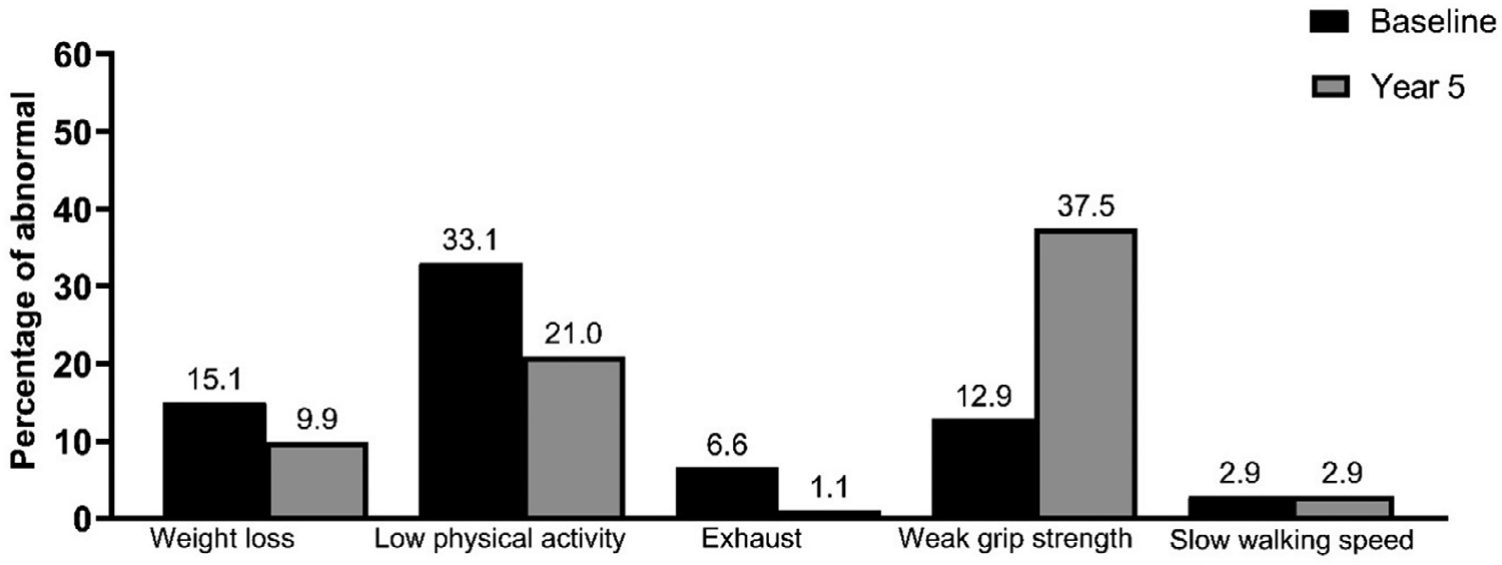
Prevalence of abnormal individual components of the Fried frailty phenotype at baseline and after 5 years of follow‐up among older adults living with HIV who completed the 5‐year follow‐up in Thailand.

At the 5‐year follow‐up, frailty prevalence increased from 13 (4.0%) to 59 (18.2%), while pre‐frailty decreased slightly from 153 (47.2%) to 141 (43.5%) (*p*<0.001) (Figure 1). Frailty prevalence was 2% among those with complete follow‐up (Figure S1). Weak grip strength became the most prevalent frailty phenotype (154, 47.2%), followed by low physical activity (109, 33.6%), and unintentional weight loss (79, 24.4%). All frailty phenotypes increased over time except low physical activity (Figure 2).

**FIGURE 3:**
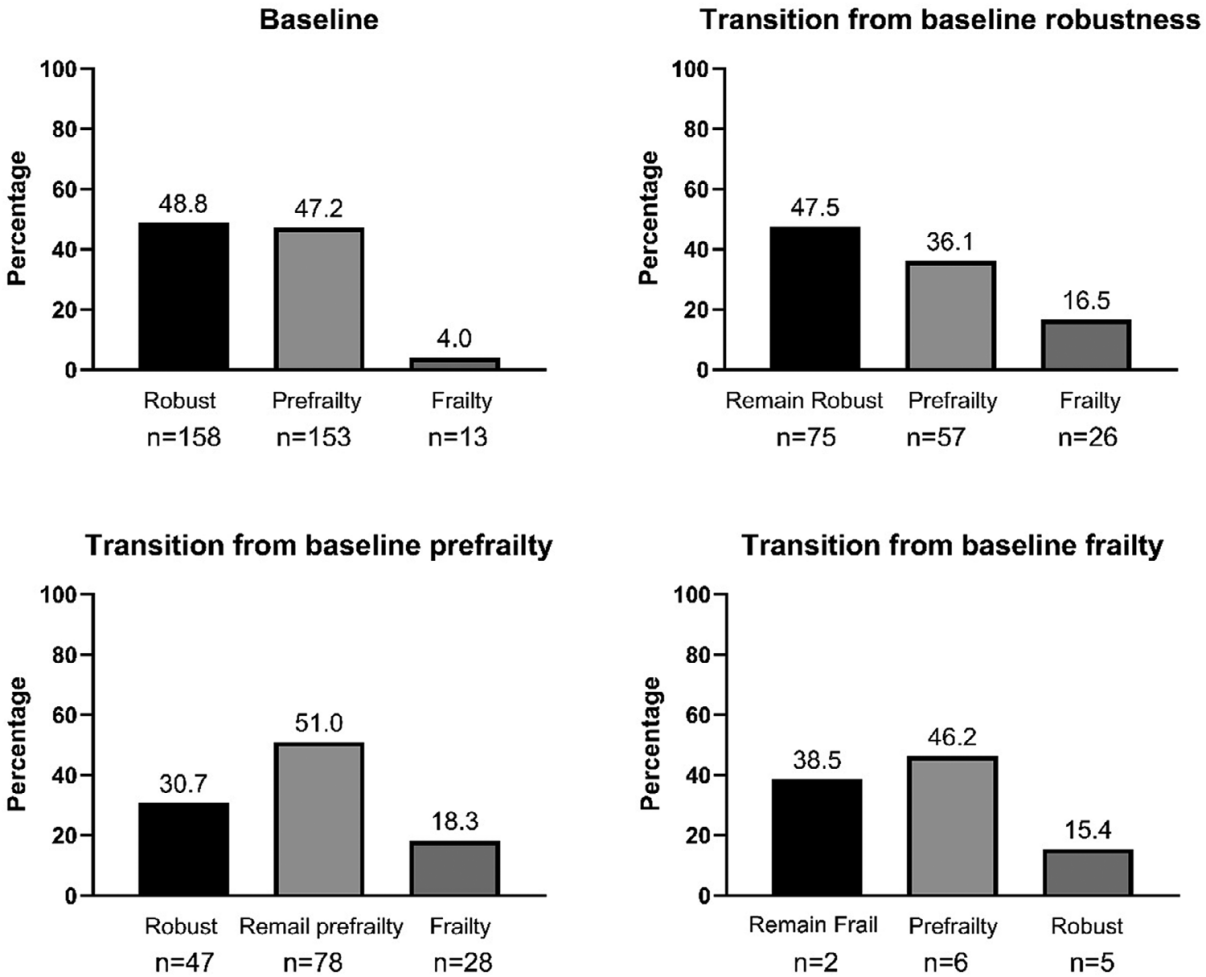
Frailty status at baseline and the transition at 5‐year visit.

Among 311 participants with baseline robust/pre‐frail status, the frailty incidence at 5 years was 17.4% (95% CI: 13.3−22.0). In 158 PWH who were initially classified as robust stage at baseline, 75 (47.5%) remained robust, 57 (36.1%) progressed to pre‐frail and 26 (16.5%) transitioned to frailty. Among 153 participants classified as pre‐frail at baseline, 28 (18.3%) progressed to frailty. Conversely, among the 13 frail participants at baseline, 38.5% (5/13) remained frail, and 61.5% (8/13) improved to prefrail or robust, including 15% (2/13) who reverted to robustness (Figure 3). Frailty transitions among participants who completed follow‐up are shown in Figure S2 and Table S9.

### 3.4 Factors Associated With Frailty

**FIGURE 4:**
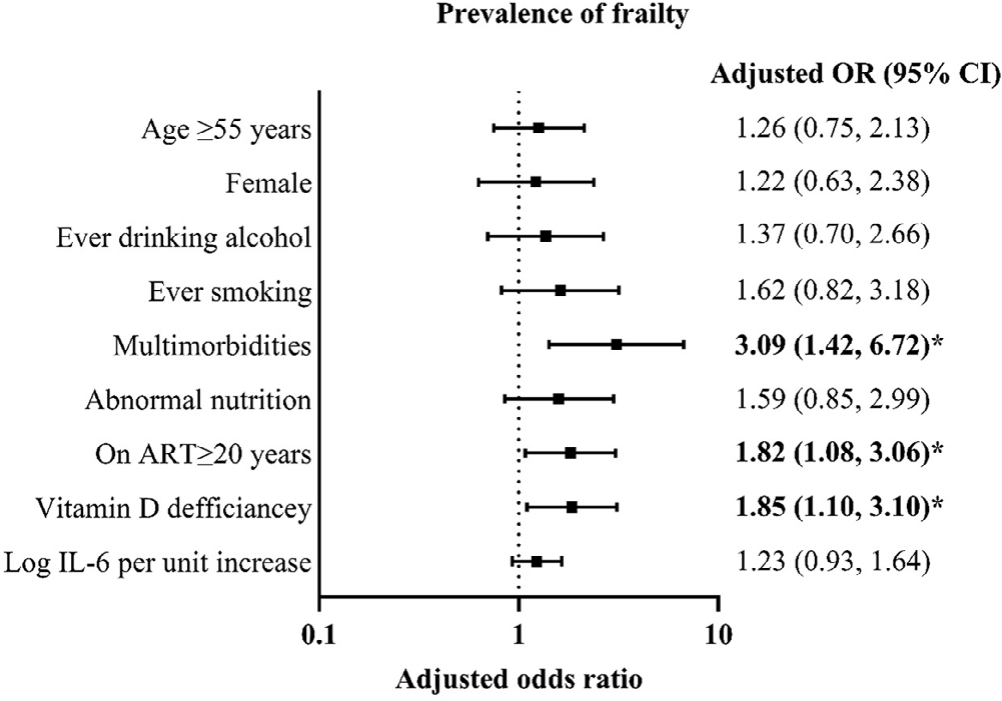
Factors associated with frailty at baseline and 5 years of follow‐up. *Note*: Frailty was modelled using a generalized estimating equation (GEE) with a binomial distribution and logit link. Adjusted odds ratios are shown on the logarithmic scale, adjusted for all variables shown. Horizontal bars indicate 95% confidence intervals. ART, antiretroviral therapy; CI, confidence interval; IL‐6, interleukin‐6; OR, odds ratio.

In the multivariate GEE analysis, multimorbidity (adjusted odds ratio [aOR]: 3.09, [95% CI: 1.42−6.72], *p* = 0.004), ART duration ≥20 years (aOR: 1.82, 95% CI: 1.08−3.06, *p* = 0.025) and baseline vitamin D deficiency (aOR: 1.85, 95% CI: 1.10−3.10, *p* = 0.019) were independently associated with increased frailty after adjusting for age, sex, alcohol, ever smoking, IL‐6 and nutrition (see Figure 4, Tables S3 and S10 for complete follow‐up).

**FIGURE 5:**
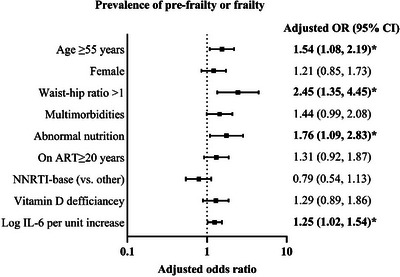
Factors associated with pre‐frailty or frailty at baseline and 5 years of follow‐up. *Note*: A generalized estimating equation (GEE) with a binomial distribution and logit link was used to model pre‐frailty or frailty status. Adjusted odds ratios are shown on the logarithmic scale, adjusted for all variables shown. Horizontal bars indicate 95% confidence intervals. ART, antiretroviral therapy; CI, confidence interval; IL‐6, interleukin‐6; NNRTI, non‐nucleoside reverse transcriptase inhibitor; OR, odds ratio.

In a second multivariate model adjusted for age, sex, multimorbidity, ART duration for ≥20 years, ART regimen and vitamin D deficiency, older age (≥55 years) (aOR: 1.54, 95% CI: 1.08−2.19, *p* = 0.016), elevated waist‐to‐hip ratio >1 (aOR: 2.45, 95% CI: 1.35−4.45, *p* = 0.003), higher baseline IL‐6 (per pg/mL increase: aOR: 1.25, 95% CI: 1.02−1.54, *p* = 0.029) and abnormal nutrition status (aOR: 1.76, 95% CI: 1.09−2.83, *p* = 0.020) were associated with pre‐frailty or frailty (see Figure 5 and Table S11 for complete follow‐up). CD4:CD8 ratio was not associated with pre‐frailty/frailty nor with frailty alone (Tables S3 and S4).

### 3.5 Factors Associated With Frailty Transition (Worsening or Reversibility)

The final model evaluating the frailty transitions included age ≥55 years, sex, ever smoking, multimorbidity, baseline CD4 <500 cell/mm^3^ and vitamin D deficiency. In multivariate analysis, factors associated with reduced likelihood of frailty improvement (i.e. frailty reversibility) were multimorbidity (aOR: 0.44, 95% CI: 0.23−0.84, *p* = 0.017) and baseline CD4 <500 cell/mm^3^ (aOR: 0.25, 95% CI: 0.10−0.62, *p* = 0.003). No factors were significantly associated with worsening frailty in the adjusted model (see Tables S5 and S6, S12 and S13 for complete follow‐up). Tables S7 and S8 compare worsening versus no worsening in participants with baseline robust/pre‐frail status and reversal versus no reversal in those with baseline pre‐frail/frail status.

## 4 Discussion

In this ageing PWH cohort, we assessed the prevalence of frailty at baseline and the transition between frailty states over a 5‐year of follow‐up. At baseline, pre‐frailty and frailty were 47% and 4%, respectively. After 5 years, frailty prevalence increased from 4.0% to 18.2%, while pre‐frailty slightly declined. When restricting analyses to participants with observed frailty assessments at the 5‐year visit, the prevalence of frailty was substantially lower (2.6%). However, 52 participants (16.0%) died or were lost to follow‐up, indicating that estimates based solely on observed data may underestimate frailty burden due to survival bias, whereas imputation approaches may overestimate frailty incidence. Together, these findings highlight the methodological challenges of estimating frailty trajectories in a long‐term cohort of ageing PWH.

Importantly, frailty was dynamic rather than static. A substantial proportion of participants demonstrated reversibility or improvement of frailty states, highlighting the potential for intervention even in older PWH.  Low physical activity and unintentional weight loss were the most common frailty components at baseline, whereas weak grip strength became the most prominent domain after 5 years of follow‐up. Additionally, the prevalence of slow walking speed increased markedly from 3.7% to 18.5%, suggesting a progressive decline in muscle strength and physical function over time. These findings underscore the muscle function as a critical target for frailty prevention and management in older PWH. Although weak grip strength and slow walking speed were common, sarcopenia remained relatively uncommon, possibly reflecting difference in the diagnostic criteria of sarcopenia applied in our study.

The prevalence of frailty among PWH varies widely across studies, largely due to heterogeneity in study populations and frailty definitions. Our findings align with prior studies using the FFP, including a systematic review and meta‐analysis reporting frailty and pre‐frailty prevalences of 10.9% and 47.2%, respectively [13]. These data confirm that frailty is common in older PWH, highlighting the importance of routine frailty screening and targeted interventions in this population. Notably, weak grip strength was the most prevalent frailty component at the 5‐year follow‐up visit, while slow walking speed showed the greatest increase from baseline. This suggests progressive declines in muscle strength and physical function as key drivers of frailty progression over time. Several studies suggest that interventions such as resistance training exercise [24], nutrition optimization and vitamin D supplement [25, 26] may help preserve muscle mass and mitigate frailty risk. Although our findings were broadly consistent with studies from high‐income settings, differences in healthcare access, socio‐economic context and population characteristics may influence the burden and presentation of frailty across settings.

In our study, multimorbidity was a key factor associated with both increased frailty risk and decreased likelihood of frailty reversal. This is consistent with previous studies reporting an increasing prevalence of frailty among participants with multimorbidity [27, 28], suggesting multimorbidity accelerates frailty progression. Achour et al. [29] also found that mitigating the burden of chronic comorbidities may improve clinical outcomes among PWH through frailty‐related pathways as well as ART‐driven virological suppression. While multimorbidity was associated with frailty, disease counts alone do not capture the severity or functional relevance of individual conditions. Consequently, management of chronic comorbidities may help reduce functional decline, treatment burden and frailty‐related outcomes, rather than decreasing the prevalence of multimorbidity. Despite high rates of virologic suppression and high current CD4 cell counts, 75% of our cohort had multimorbidity. This underscores that HIV control alone is insufficient to prevent functional decline and the need to move beyond HIV control alone towards integrated chronic comorbidities management. Although disease counts do not fully capture severity or functional impact, effective management of chronic comorbidities may reduce treatment burden, slow functional deterioration and support frailty reversal. Epidemiological evidence supports interventions targeting comorbidities assessment, smoking cessation, physical activity promotion, weight optimization, and strengthening individual and community resources to reduce frailty risk [30].

Vitamin D deficiency was independently associated with frailty in our cohort, consistent with meta‐analysis linking low serum 25‐hydroxyvitamin D (25(OH)D) levels to greater frailty severity [31, 32]. Although the benefit of vitamin D supplementation among older PWH remains controversial [33, 34, 35], vitamin D represents a potentially modifiable risk factor. Other contributors to frailty, such as sarcopenia, mitochondrial dysfunction and polypharmacy [11], further support the need for comprehensive, multidisciplinary and geriatric‐informed HIV care. Interventions such as resistance training, adequate protein intake and optimization of ART regimens can reduce mitochondrial toxicity and other adverse effects [36, 37].

Participants with baseline CD4 counts <500 cells/mm^3^ were less likely to reverse frailty, suggesting a role for premature immune senescence in ageing PWH on ART [38]. This finding is consistent with a French cohort [29] showing that lower CD4+ T‐cell counts were strongly associated with progression from pre‐frailty to frailty. Our findings on inflammatory markers align with prior studies [39, 40, 41, 42] demonstrating independent association between IL‐6 and frailty or pre‐frailty, supporting the role of chronic inflammation. This may be partly explained by the higher prevalence of multimorbidity among frailty individuals, which may elevate inflammatory markers. The lack of association between hs‐CRP and frailty may reflect limited statistical power due to the small number of frailty participants in our study.

Although longer ART duration was associated with frailty, no specific ART class was linked to frailty risk in our study. In contrast, the HAILO study [43] reported an association between NNRTI‐based regimens and severe frailty and a protective effect of INSTI regimens on gait speed. In our cohort, ART duration likely captures cumulative treatment exposure and ageing‐related factors rather than class‐specific effects, given frequent regimen changes over time.

The key strength of this study includes its longitudinal design, enabling assessment of frailty transitions and temporal relationships between risk factors and frailty over time. The comprehensive, multidimensional geriatric assessment, including multimorbidity, polypharmacy, neurocognitive impairment, sarcopenia, nutritional status and inflammatory biomarkers, provides a holistic understanding of frailty in older PWH. However, there were some limitations in our study. First, the 5‐year interval between frailty assessments led to participant attrition, and this could be because older individuals are at higher risk of becoming bedridden or dying. To mitigate this limitation, we imputed participants who were lost or died as being frail. Second, the use of only two assessment time points limited the detection of intermediate frailty transitions. Third, some frailty components relied on self‐report, although objective measures such as grip strength and gait speed were incorporated to enhance validity, this may have led to overestimation or underestimation. Lastly, ASMI was assessed using multi‐channel bioelectrical impedance analysis rather than DEXA, a pragmatic and accepted approach for large cohort studies.

## 5 Conclusions

Frailty among older PWH in Thailand is highly dynamic and potentially reversible, with over half of frail participants improving over 5 years. Multimorbidity, longer ART duration and vitamin D deficiency were associated with frailty progression, whereas higher CD4 counts and absence of multimorbidity favoured frailty reversal. These findings highlight frailty as modifiable and the need to integrate routine frailty screening, chronic disease management, nutritional support and geriatric‐informed interventions into HIV care to promote healthy ageing in resource‐limited settings.

## Author Contributions

TA, WMH, SJK and AA contributed to the design and concept of the study analysis. AH, HMSL, NH and SU conducted the frailty assessment, data collection and patient care. TA, WMH, SJK, AA and TW contributed to the analysis and interpretation of the data. TA and WMH performed statistical analysis. TA wrote the first draft of the paper. WMH, SJK, TW and AA critically reviewed the manuscript. All of the co‐authors contributed to interpreting the findings and revised the manuscript. All authors read and approved the final manuscript.

## Funding

This study was supported by the Government Research Budget year 2015 (grant code GRB‐APS‐12‐58‐30‐09), and year 2016 (grant code GRB‐APS‐04‐59‐30‐03) and Ratchadapiseksompotch Fund (grant code RA68/010). TA is supported by the Second Century Fund (C2F), Chulalongkorn University.

## Ethics Statement

This study received approval from the Ethics Committee of the Faculty of Medicine, Chulalongkorn University (approval # 0883/67). Written informed consent was obtained from all of the participants, who demonstrated understanding of the study's purpose and procedures for the main study.

## Consent

The authors have nothing to report.

## Conflicts of Interest

AA has received research grants for HIVNAT from Gilead Science, ViiV/GSK, Roche, MSD, Janssen Research & Development; transportation costs to attend meetings/conferences from Gilead Sciences; other non‐financial interests (unpaid) for working with: (1) Strategic and Technical Advisory Group to WHO for HIV/hepatitis/STI; (2) Thai AIDS Society Committee; and (3) Thailand National ART, TB, HIV, Hepatitis Program Committee. The rest of the authors declare no conflict of interest.

## Supporting information




**Table S1**: Baseline characteristics of PWH with and without frailty assessment at follow‐up visit
**Table S2**: Demographic characteristics by frailty status at 5‐year follow‐up
**Table S3**: Factors associated with frailty by using generalized estimating equation in older adults living with HIV
**Table S4**: Factors associated with prefrailty to frailty using generalized estimating equation
**Table S5**: Demographics and clinical factors by frailty transition state from baseline to 5‐year follow‐up
**Table S6**: Factors associated with worsening and improvement of the frailty stage using multinomial logistic regression
**Table S7**: Factors associated with worsening stage among participants with baseline robust/pre‐frail (*n* = 311) using logistic regression
**Table S8**: Factors associated with reversal stage among participants with baseline pre‐frail/frail (*n* = 166) using logistic regression
**Table S9**: Demographic characteristics by frailty status at 5‐year follow‐up in older adults living with HIV who completed the 5‐year follow‐up
**Table S10**: Factors associated with frailty using generalized estimating equation (GEE) in older adults living with HIV who completed the 5‐year follow‐up
**Table S11**: Factors associated with prefrailty to frailty using generalized estimating equation (GEE) in older adults living with HIV who completed the 5‐year follow‐up
**Table S12**: Associations of demographics and clinical factors by frailty transition state from baseline to 5‐year follow‐up in older adults living with HIV who completed the 5‐year follow‐up
**Table S13**: Factors associated with worsening and improvement of the frailty stage using multinomial logistic regression in older adults living with HIV who completed the 5‐year follow‐up
**Figure S1**: Prevalence of frailty at baseline and after 5 years of follow‐up in older adults living with HIV who completed the 5‐year follow‐up
**Figure S2**: Frailty status at baseline and the transition at 5 years of follow‐up among older adults living with HIV who completed the 5‐year follow‐up

## Data Availability

Data available for academics upon reasonable request from the corresponding author.
