# Supplementary material for: Frailty Transition Among Older Adults Living With HIV in Thailand: A 5‐Year Longitudinal Study
Source: J Int AIDS Soc. 2026 Apr 1;29(4):e70099. doi: 10.1002/jia2.70099 (PMC13140913; doi:10.1002/jia2.70099)

**Supplement tables**

**Table S1: Baseline characteristics of PWH with and without frailty assessment at follow-up visit**

|  | **PWH with frailty result at 5-year follow-up**  **(n=272)** | **PWH without frailty result at 5-year follow-up**  **(n=52)** | **Total**  **(n=324)** | **p-value** |
| --- | --- | --- | --- | --- |
| **Age, year** | 54.3 (51.7-59.0) | 55.6 (51.4-60.0) | 54.4 (51.7-59.3) | 0.559 |
| **Sex** |  |  |  | **0.028** |
| Male | 164 (60.3) | 40 (76.9) | 204 (63.0) |  |
| Female | 108 (39.7) | 12 (23.1) | 120 (37.0) |  |
| **Body Mass Index (BMI), kg/m^2^** | 23.1 (20.7-25.0) | 23.4 (21.7-25.6) | 23.1 (20.7-25.0) | 0.253 |
| **BMI kg/m^2^** |  |  |  | 0.391 |
| <25 | 204 (75.0) | 36 (69.2) | 240 (74.1) |  |
| ≥25 | 68 (25.0) | 16 (30.8) | 84 (25.9) |  |
| **Hepatitis B** | 29 (10.7) | 13 (25.0) | 42 (13.0) | **0.011** |
| **Hepatitis C** | 24 (8.8) | 6 (11.5) | 30 (9.3) | 0.600 |
| **Diabetes mellitus** | 41 (15.1) | 14 (26.9) | 55 (17.0) | **0.044** |
| **Hypertension** | 108 (39.7) | 28 (53.8) | 136 (42.0) | 0.066 |
| **Number of comorbidities** |  |  |  | **0.012** |
| 0 | 3 (1.1) | 0 (0.0) | 3 (0.9) |  |
| 1-2 | 107 (39.3) | 10 (19.2) | 117 (36.1) |  |
| ≥3 | 162 (59.6) | 42 (80.8) | 204 (63.0) |  |
| **Duration year on HIV** | 18.2 (15.0-20.8) | 18.9 (15.4-21.5) | 18.6 (15.0-20.9) | 0.519 |
| **Duration year on ART** | 16.1 (12.7-19.0) | 16.8 (12.9-19.2) | 16.1 (12.7-19.1) | 0.630 |
| **Current CD4, cell/mm^3^** | 621 (488-800) | 585 (400-806) | 614 (468-800) | 0.204 |
| **Nadir CD4 (cells/mm^3^)** | 176 (77-258) | 171 (86-225) | 176 (80-251) | 0.666 |
| **HIV-RNA≤50 copies/mL copies/ml** | 265 (97.4) | 50 (96.2) | 315 (97.2) | 0.641 |
| **ART regimen** |  |  |  |  |
| NNRTI-based | 153 (56.2) | 26 (50.0) | 179 (55.2) | 0.394 |
| PI-based | 89 (32.7) | 17 (32.7) | 106 (32.7) |  |
| Other | 30 (11.0) | 9 (17.3) | 39 (12.0) |  |
| **Fracture history** | 11 (4.0) | 0 (0.0) | 11 (3.4) | 0.223 |
|  |  |  |  |  |

**Note:** Categorical data are presented as frequency (percentage) and continuous data are presented as median (interquartile range)

**Abbreviations**: BMI: body mass index; PWH: people with HIV; ART: antiretroviral therapy; NNRTI: non-nucleoside reverse transcriptase inhibitor; PI: protease inhibitor

**Table S2: Demographic characteristics by frailty status at 5-yr follow-up**

|  | **Frailty status at year 5** | | | |
| --- | --- | --- | --- | --- |
|  | **Robust**  **(n= 124)** | **Prefrailty**  **(n= 141)** | **Frailty**  **(n= 59)** | **p-value** |
| **Age, year** | 54 (51-57) | 55 (52-61) | 55 (51-60) | 0.002 |
| **Sex** |  |  |  | 0.209 |
| Male | 74 (59.68) | 87 (61.70) | 43 (72.88) |  |
| Female | 50 (40.32) | 54 (38.3) | 16 (27.12) |  |
| **Ever smoked** | 44 (35.48) | 44 (31.21) | 32 (54.24) | 0.009 |
| **Ever consumed alcohol** | 21 (16.94) | 18 (12.77) | 19 (32.2) | 0.006 |
| **BMI ≥25 kg/m^2^** | 27 (21.77) | 44 (31.21) | 15 (25.42) | 0.217 |
| **Waist-hip ratio** | 0.92 (0.870.96) | 0.93 (0.890.99) | 0.94 (0.90.99) | 0.885 |
| **Metabolic syndrome** | 54 (43.55) | 77 (54.61) | 37 (62.71) | 0.036 |
| **Hepatitis B** | 7 (5.65) | 12 (8.51) | 12 (20.34) | 0.010 |
| **Hepatitis C** | 7 (5.65) | 7 (4.96) | 3 (5.08) | 1.000 |
| **Diabetes mellitus** | 37 (29.84) | 62 (43.97) | 30 (50.85) | 0.010 |
| **Hypertension** | 52 (41.94) | 81 (57.45) | 32 (54.24) | 0.037 |
| **Chronic kidney disease** | 13 (10.48) | 33 (23.40) | 19 (32.20) | 0.001 |
| **Number of comorbidities** | 3 (2-4) | 5 (3-5) | 4 (3-6) | <0.001 |
| **Multimorbidity** | 80 (64.52) | 109 (77.3) | 53 (89.83) | <0.001 |
| **Duration year on ART** | 22 (18-25) | 23 (20-25) | 21 (18-24) | 0.382 |
| **CD4 cell count (cells/mm^3^)** | 622 (488-812) | 613 (476-807) | 589 (393-748) | 0.148 |
| **HIV-RNA ≤50 copies/ml** | 120 (97.56) | 140 (99.29) | 56 (94.92) | 0.114 |
| **ART regimen** |  |  |  | <0.001 |
| NNRTI-based | 6 (4.84) | 11 (7.80) | 18 (30.51) |  |
| Other | 118 (95.16) | 130 (92.2) | 41 (69.49) |  |
| **Nutrition status** |  |  |  | 0.001 |
| Normal | 117 (94.35) | 119 (84.40) | 45 (76.27) |  |
| Abnormal | 7 (5.65) | 22 (15.60) | 14 (23.73) |  |
| **Depression** | 59 (47.58) | 75 (53.57) | 3 (42.86) | 0.644 |
| **ASCVD risk score** | 8.5 (4.4-15.3) | 12.7 (8.0-23.9) | 15.0 (8.0-23.2) | <0.001 |
| **Cognitive impairment** | 86 (69.35) | 91 (64.54) | 37 (63.79) | 0.643 |
| **Number of drugs include ART** | 5 (4-6) | 5 (4-7) | 5 (4-8) | 0.094 |
| **Fracture history** | 1 (0.81) | 7 (4.96) | 1 (1.69) | 0.099 |

**Note:** Categorical data are presented as frequency (percentage) and continuous data are presented as median (interquartile range)

**Abbreviations**: BMI: body mass index; ASCVD: atherosclerotic cardiovascular disease; ART: antiretroviral therapy; NNRTI: non-nucleoside reverse transcriptase inhibitor; PI: protease inhibitor

**Table S3: Factors associated with frailty by using generalized estimating equation in older adults living with HIV**

| **Characteristics** | **Univariable** | | | **Multivariable** | | |
| --- | --- | --- | --- | --- | --- | --- |
|  | **OR** | **95%CI** | **p-value** | **Adj OR** | **95%CI** | **p-value** |
| Age ≥ 55 year | 1.43 | 0.87-2.35 | 0.162 | 1.26 | 0.75-2.13 | 0.382 |
| Female (vs. male) | 0.83 | 0.49-1.41 | 0.497 | 1.22 | 0.63-2.38 | 0.553 |
| Waist-hip ratio > 1 (vs <1) | 1.34 | 0.65-2.75 | 0.433 |  |  |  |
| Ever consumed alcohol (vs. not drink) | 1.81 | 1.04-3.15 | 0.035 | 1.37 | 0.70-2.66 | 0.359 |
| Ever Smoked (vs. never) | 1.78 | 1.08-2.92 | 0.024 | 1.62 | 0.82-3.18 | 0.164 |
| Hypertension | 1.51 | 0.92-2.47 | 0.103 |  |  |  |
| Diabetes mellitus | 2.39 | 1.45-3.95 | 0.001 |  |  |  |
| Hepatitis B | 2.12 | 1.10-4.07 | 0.025 |  |  |  |
| Chronic kidney diseases | 3.61 | 2.09-6.26 | <0.001 |  |  |  |
| Multimorbidity | 4.06 | 1.91-8.64 | <0.001 | 3.09 | 1.42-6.72 | 0.004 |
| ASCVD ≥5% vs (<5) | 2.31 | 1.23-4.33 | 0.009 |  |  |  |
| Cognitive impairment | 1.16 | 0.69-1.97 | 0.568 |  |  |  |
| Abnormal nutrition (vs. normal) | 1.78 | 0.98-3.23 | 0.056 | 1.59 | 0.85-2.99 | 0.150 |
| Baseline osteoporosis vs (normal) | 1.01 | 0.49-2.10 | 0.974 |  |  |  |
| Fracture history (vs none) | 0.89 | 0.20-3.98 | 0.874 |  |  |  |
| Duration year on ART ≥20 (vs. <20) | 1.94 | 1.19-3.17 | 0.008 | 1.82 | 1.08-3.06 | 0.025 |
| ART regimen |  |  |  |  |  |  |
| NNRTI-based | 1 |  |  |  |  |  |
| PI-based/ INSTI-based/ Others | 1.08 | 0.64-1.83 | 0.772 |  |  |  |
| Current CD4 cell count < 500 cells/mm^3^ | 1.31 | 0.77-2.2 | 0.318 |  |  |  |
| Log baseline IL-6 (pg/ml) per a unit increase | 1.39 | 1.06-1.83 | 0.019 | 1.23 | 0.93-1.64 | 0.142 |
| Baseline hs-CRP (mg/L) per a unit increase | 0.99 | 0.95-1.04 | 0.805 |  |  |  |
| Vitamin D deficiency (<20) (vs. ≥20) | 1.97 | 1.19-3.28 | 0.009 | 1.85 | 1.10-3.10 | 0.019 |
| CD4/CD8 ratio, per a unit increase | 0.93 | 0.59-1.48 | 0.770 |  |  |  |

**Note:** Frailty was modeled using a generalized estimating equation (GEE) with a binomial distribution and logit link. Multimorbidity was used instead of individual comorbidities to avoid multicollinearity.

**Abbreviations**: BMI: body mass index; ASCVD: atherosclerotic cardiovascular disease; ART: antiretroviral therapy; NNRTI: non-nucleoside reverse transcriptase inhibitor; PI: protease inhibitor; INSTI: integrase stand transfer inhibitors; IL-6: Interleukin-6; hs-CRP: high sensitivity CRP; OR: odds ratio; Adj OR: adjusted odds ratio

**Table S4: Factor associated with prefrailty to frailty by using generalized estimating equation**

| **Characteristics** | **Univariable** | | | | **Multivariable** | | |
| --- | --- | --- | --- | --- | --- | --- | --- |
|  | **OR** | **95%CI** | **p-value** | **Adj OR** | | **95%CI** | **p-value** |
| Age≥ 55 year | 1.57 | 1.12-2.20 | 0.009 | 1.54 | | 1.08-2.19 | 0.016 |
| Female (vs. male) | 1.08 | 0.76-1.53 | 0.671 | 1.21 | | 0.85-1.73 | 0.292 |
| Waist-hip ratio > 1 (vs <1) | 2.49 | 1.41-4.37 | 0.002 | 2.45 | | 1.35-4.45 | 0.003 |
| Ever consumed alcohol (vs. not drink) | 0.92 | 0.62-1.37 | 0.676 |  | |  |  |
| Ever Smoked (vs. never) | 1.03 | 0.73-1.45 | 0.850 |  | |  |  |
| Hypertension | 1.68 | 1.21-2.34 | 0.002 |  | |  |  |
| Diabetes mellitus | 1.68 | 1.17-2.42 | 0.005 |  | |  |  |
| Hepatitis B | 1.14 | 0.67-1.93 | 0.635 |  | |  |  |
| Chronic kidney diseases | 2.64 | 1.59-4.37 | <0.001 |  | |  |  |
| Multimorbidity | 1.77 | 1.25-2.50 | 0.001 | 1.44 | | 0.99-2.08 | 0.053 |
| ASCVD ≥5% vs (<5) | 1.41 | 1.00-1.98 | 0.051 |  | |  |  |
| Cognitive impairment | 1.05 | 0.76-1.46 | 0.766 |  | |  |  |
| Abnormal nutrition (vs. normal) | 1.78 | 1.13-2.81 | 0.013 | 1.76 | | 1.09-2.83 | 0.020 |
| Baseline osteoporosis vs (normal) | 1.05 | 0.65-1.70 | 0.843 |  | |  |  |
| Fracture history (vs none) | 2.33 | 0.78-6.94 | 0.129 |  | |  |  |
| Duration year on ART ≥ 20 (vs. <20) | 1.46 | 1.07-1.99 | 0.018 | 1.31 | | 0.92-1.87 | 0.137 |
| ART regimen |  |  |  |  | |  |  |
| NNRTI-based | 1 |  |  |  | |  |  |
| PI-based/ INSTI-based/ Others | 1.40 | 1.02-1.93 | 0.039 | 1.27 | | 0.88-1.84 | 0.197 |
| Current CD4 cell count < 500 cells/mm^3^ | 0.85 | 0.60-1.21 | 0.375 |  | |  |  |
| Log baseline IL-6 (pg/ml) per a unit increase | 1.27 | 1.05-1.55 | 0.016 | 1.25 | | 1.02-1.54 | 0.029 |
| Baseline hs-CRP, (mg/L) per a unit increase | 0.99 | 0.97-1.02 | 0.704 |  | |  |  |
| Vitamin D deficiency (<20) (vs. ≥20) | 1.27 | 0.89-1.81 | 0.191 | 1.29 | | 0.89-1.86 | 0.175 |
| CD4/CD8 ratio, per a unit increase | 1.00 | 0.74-1.35 | 0.998 |  | |  |  |

**Note:** A generalized estimating equation (GEE) with a binomial distribution and logit link was used to model pre-frailty or frailty status. Multimorbidity was used instead of individual comorbidities to avoid multicollinearity.

**Abbreviations**: PWH: people with HIV; ASCVD: atherosclerotic cardiovascular disease; ART: antiretroviral therapy; NNRTI: non-nucleoside reverse transcriptase inhibitor; PI: protease inhibitor; INSTI: integrase stand transfer inhibitors; IL-6: Interleukin-6; CRP: C-reactive protein; hs-CRP: high sensitivity CRP; OR: odds ratio; Adj OR: adjusted odds ratio; CI: confidence interval

**Table S5: Demographics and clinical factors by Frailty transition state from baseline to 5-year follow-up**

|  | **Frailty transition state from baseline** | | | |
| --- | --- | --- | --- | --- |
|  | **Stable**  **(n=158)** | **Increase**  **(n=111)** | **Decrease**  **(n=55)** | **p-value** |
| Age |  |  |  | 0.803 |
| Age< 55 year | 83 (52.5) | 54 (48.6) | 28 (50.9) |  |
| Age≥ 55 year | 75 (47.5) | 57 (51.4) | 27 (49.1) |  |
| Sex |  |  |  | 0.03 |
| Male | 99 (62.7) | 78 (70.3) | 27 (49.1) |  |
| Female | 59 (37.3) | 33 (29.7) | 28 (50.9) |  |
| Waist-hip ratio |  |  |  | 0.224 |
| < 1 | 146 (92.4) | 98 (88.3) | 53 (96.4) |  |
| >1 | 12 (7.6) | 13 (11.7) | 2 (3.6) |  |
| Ever consumed alcohol |  |  |  | 0.221 |
| No | 126 (79.7) | 82 (73.9) | 47 (85.5) |  |
| Yes | 32 (20.3) | 29 (26.1) | 8 (14.5) |  |
| Ever Smoked |  |  |  | 0.042 |
| No | 106 (67.1) | 62 (55.9) | 41 (74.5) |  |
| Yes | 52 (32.9) | 49 (44.1) | 14 (25.5) |  |
| Hypertension | 70 (44.3) | 49 (44.1) | 17 (30.9) | 0.193 |
| Diabetes mellitus | 29 (18.4) | 24 (21.6) | 2 (3.6) | 0.006 |
| Hepatitis B | 18 (11.4) | 19 (17.1) | 5 (9.1) | 0.269 |
| Hepatitis C | 19 (12.0) | 8 (7.20) | 3 (5.50) | 0.261 |
| Chronic kidney diseases | 13 (8.20) | 11 (9.90) | 5 (9.10) | 0.869 |
| Multimorbidity | 101 (63.9) | 79 (71.2) | 24 (43.6) | 0.003 |
| ASCVD ≥5% vs (<5) | 93 (58.9) | 74 (66.7) | 23 (41.8) | 0.010 |
| Cognitive impairment | 93 (58.9) | 66 (60.0) | 34 (63.0) | 0.868 |
| Abnormal nutrition | 28 (17.7) | 23 (20.7) | 8 (14.5) | 0.628 |
| Baseline osteoporosis | 21 (13.8) | 17 (16.2) | 8 (14.8) | 0.848 |
| Fracture history | 5 (3.2) | 4 (3.6) | 2 (3.6) | 1.00 |
| Duration year on ART ≥ 20 | 14 (8.9) | 13 (11.7) | 8 (14.5) | 0.444 |
| ART regimen |  |  |  | 0.378 |
| NNRTI-based | 88 (55.7) | 65 (58.6) | 26 (47.3) |  |
| PI-based/ INSTI-based/ Others | 70 (44.3) | 46 (41.4) | 29 (52.7) |  |
| Current CD4 cell count < 500 cells/mm^3^ | 55 (34.8) | 35 (31.5) | 6 (10.9) | 0.002 |
| Baseline IL-6 (pg/ml) | 5.8 (4.2-7.6) | 6.3 (3.6-9.2) | 5.9 (4.6-8.0) | 0.784 |
| Baseline hs-CRP ≥2, (mg/L) | 46 (29.1) | 40 (36.0) | 24 (43.6) | 0.127 |
| Vitamin D deficiency (<20) (vs. ≥20) | 51 (32.7) | 47 (43.5) | 15 (27.3) | 0.079 |

**Note:** Categorical data are presented as frequency (percentage) and continuous data are presented as median (interquartile range)

**Abbreviations**: ASCVD: atherosclerotic cardiovascular disease; ART: antiretroviral therapy; NNRTI: non-nucleoside reverse transcriptase inhibitor; PI: protease inhibitor; INSTI: integrase stand transfer inhibitors; IL-6: Interleukin-6; CRP: C-reactive protein; hs-CRP: high sensitivity CRP

**Table S6: Factors associated with worsening and improvement frailty stage by using multinomial logistic regression**

|  | **Outcome** | | **Outcome** | |
| --- | --- | --- | --- | --- |
|  | **worsened vs stable** | | **improved vs stable** | |
|  | **OR (95%CI)** | **Adj OR (95%CI)** | **OR (95%CI)** | **Adj OR (95%CI)** |
| Age≥ 55 year | 1.17 (0.72-1.90) | 1.15 (0.69-1.92) | 1.07 (0.58-1.97) | 1.27 (0.66-2.45) |
| Female (vs. male) | 0.71 (0.42-1.19) | 0.80 (0.44-1.48) | 1.74 (0.94-3.23) | 1.60 (0.75-3.41) |
| Waist-hip ratio > 1 (vs <1) | 1.61 (0.71-3.68) |  | 0.46 (0.10-2.12) |  |
| Ever consumed alcohol (vs. not drink) | 1.39 (0.78-2.47) |  | 0.67 (0.29-1.56) |  |
| Ever Smoked (vs. never) | 1.61 (0.98-2.66) | 1.52 (0.85-2.73) | 0.70 (0.35-1.39) | 1.10 (0.47-2.55) |
| Hypertension | 0.99 (0.61-1.62) |  | 0.56 (0.29-1.08) |  |
| Diabetes mellitus | 1.23 (0.67-2.25) |  | 0.17 (0.04-0.73) |  |
| Multimorbidity | 1.39 (0.83-2.35) | 1.27 (0.73-2.19) | 0.44 (0.23-0.82) | **0.44 (0.23-0.84)** |
| Abnormal nutrition (vs. normal) | 1.21 (0.66-2.24) |  | 0.79 (0.34-1.86) |  |
| Duration year on ART ≥ 20 | 1.36 (0.61-3.03) |  | 1.75 (0.69-4.43) |  |
| Current CD4 cell count < 500 cells/mm^3^ | 0.86 (0.51-1.45) | 0.79 (0.46-1.35) | 0.23 (0.09-0.57) | **0.25 (0.10-0.62)** |
| Log baseline IL-6 (pg/ml) per a unit increase | 0.88 (0.67-1.16) |  | 1.15 (0.81-1.63) |  |
| Baseline hs-CRP ≥ 2, (mg/L) | 1.37 (0.82-2.30) |  | 1.88 (1.00-3.55) |  |
| Vitamin D deficiency (<20) (vs. ≥20) | 1.59 (0.96-2.63) | 1.62 (0.97-2.73) | 0.77 (0.39-1.53) | 0.90 (0.44-1.83) |

**Note:** Factors associated with worsening and improvement in frailty stage were examined using multinomial logistic regression. Multimorbidity was used instead of individual comorbidities to avoid multicollinearity.

**Abbreviations**: ART: antiretroviral therapy; IL-6: Interleukin-6; CRP: C-reactive protein; hs-CRP: high sensitivity CRP; OR: odds ratio; Adj OR: adjusted odds ratio; CI: confidence interval

**Table S7: Factors associated with worsening stage among participants with baseline robust/pre-frail (n=311), using logistic regression**

| **Baseline characteristics** | **Univariable** | | | **Multivariable** | | |
| --- | --- | --- | --- | --- | --- | --- |
|  | **OR** | **95%CI** | **p-value** | **Adj OR** | **95%CI** | **p-value** |
| Age ≥ 55 year | 1.21 | 0.76-1.93 | 0.412 | 1.10 | 0.67-1.82 | 0.703 |
| Female (vs. male) | 0.65 | 0.39-1.06 | 0.086 | 0.84 | 0.46-1.54 | 0.576 |
| Waist-hip ratio > 1 (vs <1) | 1.60 | 0.79-3.26 | 0.192 | 1.29 | 0.60-2.79 | 0.512 |
| Ever consumed alcohol (vs. not drink) | 1.51 | 0.87-2.62 | 0.144 | 0.97 | 0.49-1.90 | 0.924 |
| Ever Smoked (vs. never) | 1.72 | 1.06-2.77 | 0.027 | 1.48 | 0.79-2.76 | 0.217 |
| Multimorbidity | 1.85 | 1.14-3.01 | 0.013 | 1.57 | 0.92-2.66 | 0.096 |
| Cognitive impairment | 1.05 | 0.65-1.69 | 0.836 |  |  |  |
| Abnormal nutrition (vs. normal) | 1.32 | 0.73-2.39 | 0.354 |  |  |  |
| Baseline osteoporosis vs (normal) | 1.19 | 0.61-2.30 | 0.609 |  |  |  |
| Fracture history (vs none) | 1.21 | 0.33-4.38 | 0.773 |  |  |  |
| Duration year on ART ≥20 (vs. <20) | 1.19 | 0.57-2.50 | 0.639 |  |  |  |
| ART regimen |  |  |  |  |  |  |
| NNRTI-based | 1.39 | 0.82-2.36 | 0.219 |  |  |  |
| INSTI-based/ Others | 1.78 | 0.82-3.87 | 0.147 |  |  |  |
| Current CD4 cell count < 500 cells/mm^3^ | 1.13 | 0.68-1.87 | 0.640 |  |  |  |
| Log baseline IL-6 (pg/ml) per a unit increase | 0.88 | 0.68-1.14 | 0.334 |  |  |  |
| Vitamin D deficiency (<20) (vs. ≥20) | 1.73 | 1.06-2.81 | 0.027 | 1.74 | 1.05-2.90 | **0.032** |
| CD4/CD8 ratio, per a unit increase | 0.61 | 0.35-1.04 | 0.070 | 0.71 | 0.40-1.25 | 0.233 |

**Note:** Logistic regression analysis of factors associated with worsening frailty stage among participants robust or pre-frail at baseline. Multimorbidity was used instead of individual comorbidities to avoid multicollinearity.

**Abbreviations**: ART: antiretroviral therapy; IL-6: Interleukin-6; OR: odds ratio; Adj OR: adjusted odds ratio; CI: confidence interval; ART: antiretroviral therapy; NNRTI: non-nucleoside reverse transcriptase inhibitor; INSTI: integrase stand transfer inhibitors

**Table S8: Factors associated with reversal stage among participants with baseline pre-frail/frail (n=166), using logistic regression**

| **Baseline characteristics** | **Univariable** | | | **Multivariable** | | |
| --- | --- | --- | --- | --- | --- | --- |
|  | **OR** | **95%CI** | **p-value** | **Adj OR** | **95%CI** | **p-value** |
| Age ≥ 55 year | 0.76 | 0.4-1.46 | 0.411 | 0.97 | 0.47-2.00 | 0.942 |
| Female (vs. male) | 1.77 | 0.92-3.41 | 0.087 | 1.72 | 0.85-3.48 | 0.132 |
| Waist-hip ratio > 1 (vs <1) | 0.28 | 0.06-1.31 | 0.106 |  |  |  |
| Ever consumed alcohol (vs. not drink) | 0.59 | 0.24-1.4 | 0.229 |  |  |  |
| Ever Smoked (vs. never) | 0.54 | 0.26-1.11 | 0.092 |  |  |  |
| Multimorbidity | 0.24 | 0.12-0.47 | <0.001 | 0.29 | 0.14-0.60 | **0.001** |
| Cognitive impairment | 0.97 | 0.49-1.91 | 0.933 |  |  |  |
| Abnormal nutrition (vs. normal) | 0.56 | 0.23-1.33 | 0.186 |  |  |  |
| Baseline osteoporosis vs (normal) | 0.98 | 0.39-2.45 | 0.963 |  |  |  |
| Fracture history (vs none) | 0.80 | 0.15-4.26 | 0.794 |  |  |  |
| Duration year on ART ≥20 (vs. <20) | 1.55 | 0.58-4.10 | 0.380 |  |  |  |
| ART regimen |  |  |  |  |  |  |
| NNRTI-based | Ref |  |  |  |  |  |
| INSTI-based/ Others | 1.06 | 0.55-2.02 | 0.867 |  |  |  |
| Current CD4 cell count < 500 cells/mm^3^ | 0.28 | 0.11-0.71 | 0.007 | 0.36 | 0.13-0.97 | **0.044** |
| Log baseline IL-6 (pg/ml) per a unit increase | 0.96 | 0.66-1.4 | 0.832 |  |  |  |
| Vitamin D deficiency (<20) (vs. ≥20) | 0.55 | 0.27-1.12 | 0.101 | 0.75 | 0.34-1.62 | 0.460 |
| CD4/CD8 ratio, per a unit increase | 1.89 | 0.96-3.72 | 0.067 |  |  |  |

**Note:** Logistic regression analysis of factors associated with reversal frailty stage among participants with pre-frail/frail at baseline. Multimorbidity was used instead of individual comorbidities to avoid multicollinearity.

**Abbreviations**: OR: odds ratio; Adj OR: adjusted odds ratio; CI: confidence interval; ART: antiretroviral therapy; NNRTI: non-nucleoside reverse transcriptase inhibitor; INSTI: integrase stand transfer inhibitors; IL-6: Interleukin-6

**Table S9: Demographic characteristics by frailty status at 5-yr follow-up in older adults living with HIV who** **completed the 5-year follow-up**

|  | **Frailty status at year 5** | | | |
| --- | --- | --- | --- | --- |
|  | **Robust**  **(n= 125)** | **Robust**  **(n= 140)** | **Robust**  **(n= 7)** | **p-value** |
| **Age, year** | 60 (57-63) | 61 (59-67) | 61 (58-66) | 0.003 |
| **Sex** |  |  |  | 0.578 |
| Male | 74 (59.2) | 87 (62.1) | 3 (42.9) |  |
| Female | 51 (40.8) | 53 (37.9) | 4 (57.1) |  |
| **Ever smoked** | 44 (35.5) | 44 (31.2) | 3 (42.9) | 0.655 |
| **Ever consumed alcohol** | 21 (16.9) | 18 (12.8) | 1 (14.3) | 0.590 |
| **Waist-hip ratio > 1** | 6 (4.8) | 31 (22.14) | 1 (14.29) | <0.001 |
| **Metabolic syndrome** | 55 (44.0) | 75 (53.6) | 6 (85.7) | 0.049 |
| **Hepatitis B** | 7 (5.6) | 12 (8.6) | 0 (0.0) | 0.686 |
| **Hepatitis C** | 7 (5.6) | 7 (5.0) | 0 (0.0) | 1.000 |
| **Diabetes mellitus** | 38 (30.4) | 61 (43.6) | 5 (71.4) | 0.014 |
| **Hypertension** | 52 (41.6) | 81 (57.9) | 4 (57.1) | 0.030 |
| **Chronic kidney disease** | 13 (10.4) | 33 (23.6) | 4 (57.1) | <0.001 |
| **Number of comorbidities** | 3 (2-4) | 5 (3-5) | 4 (4-5) | <0.001 |
| **Multimorbidity** | 80 (64.5) | 109 (77.3) | 7 (100.0) | 0.015 |
| **Duration year on ART** | 22 (18-24) | 23 (20-25) | 20 (17-22) | 0.119 |
| **CD4 cell count (cells/mm^3^)** | 615 (497-812) | 608 (475-808) | 748 (547-780) | 0.658 |
| **HIV-RNA ≤50 copies/ml** | 121 (97.6) | 139 (99.3) | 7 (100.0) | 0.410 |
| **ART regimen** |  |  |  | 0.576 |
| NNRTI-based | 6 (4.8) | 11 (7.9) | 0 (0.0) |  |
| Others | 119 (95.2) | 129 (92.1) | 7 (100.0) |  |
| **Nutrition status** |  |  |  | 0.002 |
| Normal | 118 (94.4) | 117 (84.2) | 4 (57.1) |  |
| Abnormal | 7 (5.6) | 22 (15.8) | 3 (42.9) |  |
| **Depression** | 59 (47.6) | 75 (53.6) | 3 (42.9) | 0.644 |
| **ASCVD risk score** | 8.4 (4.4-15.3) | 12.7 (7.8-25.2) | 15.0 (7.7-23.2) | <0.001 |
| **Cognitive impairment** | 87 (69.6) | 89 (64.0) | 4 (66.7) | 0.632 |
| **Number of medications including ART** | 5 (4-6) | 5 (4-7) | 10 (4-12) | 0.007 |
| **Fracture history** | 1 (0.8) | 7 (5.0) | 1 (14.3) | 0.035 |

**Note:** Categorical data are presented as frequency (percentage) and continuous data are presented as median (interquartile range)

**Abbreviations**: ASCVD: atherosclerotic cardiovascular disease; ART: antiretroviral therapy; NNRTI: non-nucleoside reverse transcriptase inhibitor

**Table S10: Factors associated with frailty by using generalized estimating equation (GEE) in older adults living with HIV who** **completed the 5-year follow-up**

| **Characteristics** | **Univariate** | | | | **Multivariable** | | |
| --- | --- | --- | --- | --- | --- | --- | --- |
|  | **OR** | **95%CI** | **p-value** | **Adj OR** | | **95%CI** | **p-value** |
| Age ≥ 55 year | 1.61 | 0.66, 3.97 | 0.299 | 1.28 | | 0.51, 3.21 | 0.597 |
| Female (vs. male) | 2.51 | 1.02, 6.19 | 0.046 | 2.73 | | 1.10, 6.79 | 0.031 |
| Waist-hip ratio > 1 (vs <1) | 1.48 | 0.43-5.16 | 0.536 |  | |  |  |
| Ever drink alcohol (vs. Not drink) | 0.79 | 0.23, 2.72 | 0.705 |  | |  |  |
| Ever Smoking (vs. never) | 0.8 | 0.30, 2.11 | 0.651 |  | |  |  |
| Multimorbidity | 4.63 | 1.07, 20.10 | 0.041 | 4.83 | | 1.09, 21.39 | 0.038 |
| Cognitive impairment | 1.67 | 0.59-4.71 | 0.329 |  | |  |  |
| Abnormal nutrition (vs. normal) | 2.48 | 0.93, 6.62 | 0.070 | 2.42 | | 0.89, 6.55 | 0.082 |
| Baseline osteoporosis vs (normal) | 1.45 | 0.48, 4.40 | 0.509 |  | |  |  |
| Fracture history (vs none) | 3.46 | 0.76, 15.78 | 0.109 |  | |  |  |
| Duration on ART > 20 year | 0.74 | 0.28, 1.96 | 0.544 |  | |  |  |
| ARV regimen |  |  |  |  | |  |  |
| NNRTI-based | 0.67 | 0.24, 1.88 | 0.450 |  | |  |  |
| PI-based/ INSTI-based/ Others | Ref |  |  |  | |  |  |
| Current CD4 < 500 cell/mm^3^ | 0.60 | 0.20, 1.82 | 0.368 |  | |  |  |
| Baseline IL-6 (pg/ml) per a unit increase | 1.00 | 0.98, 1.02 | 0.891 |  | |  |  |
| High sensitivity CRP (mg/dl) per a unit increase | 0.99 | 0.90, 1.08 | 0.778 |  | |  |  |
| Vitamin D deficiency (<20) (vs. >20) | 1.77 | 0.71, 4.41 | 0.217 |  | |  |  |

**Note:** Frailty was modeled using a generalized estimating equation (GEE) with a binomial distribution and logit link in available population. Multimorbidity was used instead of individual comorbidities to avoid multicollinearity.

**Abbreviations**: OR: odds ratio; Adj OR: adjusted odds ratio; CI: confidence interval; ART: antiretroviral therapy; NNRTI: non-nucleoside reverse transcriptase inhibitor; INSTI: integrase stand transfer inhibitors; PI: protease inhibitor; CRP: C-reactive protein; hs-CRP: high sensitivity CRP, IL-6: Interleukin-6

**Table S11: Factors associated with prefrailty to frailty by using generalized estimating equation (GEE) in older adults living with HIV who** **completed the 5-year follow-up**

| **Characteristics** | **Univariate** | | | | **Multivariable** | | |
| --- | --- | --- | --- | --- | --- | --- | --- |
|  | **OR** | **95%CI** | **p-value** | **Adj OR** | | **95%CI** | **p-value** |
| Age≥ 55 year | 1.54 | 1.08, 2.19 | 0.016 | 1.39 | | 0.97, 2.00 | 0.071 |
| Female (vs. male) | 1.20 | 0.84, 1.72 | 0.323 | 1.21 | | 0.84, 1.75 | 0.309 |
| Waist-hip ratio > 1 (vs <1) | 2.51 | 1.41, 4.45 | 0.002 |  | |  |  |
| Ever drink alcohol (vs. Not drink) | 0.80 | 0.52, 1.21 | 0.286 |  | |  |  |
| Ever Smoking (vs. never) | 0.91 | 0.64, 1.30 | 0.610 |  | |  |  |
| Multimorbidity | 1.54 | 1.08, 2.19 | 0.018 | 1.27 | | 0.87, 1.84 | 0.217 |
| Cognitive impairment | 1.02 | 0.73-1.43 | 0.908 |  | |  |  |
| Abnormal nutrition (vs. normal) | 1.72 | 1.08, 2.73 | 0.022 | 1.98 | | 1.22, 3.19 | 0.005 |
| Baseline osteoporosis vs (normal) | 1.08 | 0.65, 1.77 | 0.775 |  | |  |  |
| Fracture history (vs none) | 2.72 | 0.91, 8.14 | 0.074 | 2.57 | | 0.84, 7.92 | 0.100 |
| Duration on ART > 20 year | 1.25 | 0.91, 1.73 | 0.173 |  | |  |  |
| ARV regimen |  |  |  |  | |  |  |
| NNRTI-based | 0.71 | 0.51, 0.98 | 0.039 | 0.68 | | 0.48, 0.96 | 0.028 |
| PI-based/ INSTI-based/ Others | Ref |  |  | Ref | |  |  |
| Current CD4 < 500 cell/mm^3^ | 0.78 | 0.54, 1.14 | 0.201 |  | |  |  |
| Baseline IL-6 (pg/ml) per a unit increase | 1.01 | 1.00, 1.02 | 0.253 |  | |  |  |
| High sensitivity CRP, (mg/dl) per a unit increase | 1.00 | 0.97, 1.02 | 0.717 |  | |  |  |
| Vitamin D deficiency (<20) (vs. >20) | 1.16 | 0.80, 1.69 | 0.422 |  | |  |  |

**Note:** A generalized estimating equation (GEE) with a binomial distribution and logit link was used to model pre-frailty or frailty status in available population. Multimorbidity was used instead of individual comorbidities to avoid multicollinearity.

**Abbreviations**: OR: odds ratio; Adj OR: adjusted odds ratio; CI: confident interval; ART: antiretroviral therapy; NNRTI: non-nucleoside reverse transcriptase inhibitor; INSTI: integrase stand transfer inhibitors; PI: protease inhibitor; CRP: C-reactive protein; hs-CRP: high sensitivity CRP; IL-6: Interleukin-6

**Table S12: Associations of demographics and clinical factors by Frailty transition state from baseline to 5-year follow-up in older adults living with HIV who** **completed the 5-year follow-up**

|  | **Frailty transition state from baseline** | | | |
| --- | --- | --- | --- | --- |
|  | **Stable**  **N=153** | **Increase**  **N=64** | **Decrease**  **N=55** | **p-value** |
| Age |  |  |  | 0.855 |
| Age< 55 year | 82 (53.6) | 32 (50.0) | 28 (50.9) |  |
| Age≥ 55 year | 71 (46.4) | 32 (50.0) | 27 (49.1) |  |
| Sex |  |  |  | 0.166 |
| Male | 96 (62.7) | 41 (64.1) | 27 (49.1) |  |
| Female | 57 (37.3) | 23 (35.9) | 28 (50.9) |  |
| Waist-hip ratio |  |  |  | 0.210 |
| < 1 | 142 (92.81) | 56 (87.5) | 53 (96.36) |  |
| >1 | 11 (7.19) | 8 (12.5) | 2 (3.64) |  |
| Ever drink alcohol |  |  |  | 0.690 |
| No | 123 (80.4) | 51 (79.7) | 47 (85.5) |  |
| Yes | 30 (19.6) | 13 (20.3) | 8 (14.5) |  |
| Ever Smoking |  |  |  | 0.455 |
| No | 104 (68.0) | 41 (64.1) | 41 (74.5) |  |
| Yes | 49 (32.0) | 23 (35.9) | 14 (25.5) |  |
| Hypertension | 65 (42.5) | 26 (40.6) | 17 (30.9) | 0.328 |
| Diabetes mellitus | 26 (17.0) | 13 (20.3) | 2 (3.6) | 0.013 |
| Hepatitis B | 18 (11.8) | 6 (9.4) | 5 (9.1) | 0.895 |
| Hepatitis C | 18 (11.8) | 3 (4.7) | 3 (5.5) | 0.183 |
| Chronic kidney diseases | 10 (6.5) | 4 (6.2) | 5 (9.1) | 0.805 |
| Multimorbidity | 96 (62.7) | 42 (65.6) | 24 (43.6) | 0.026 |
| ASCVD ≥5% vs (<5) | 89 (58.2) | 40 (62.5) | 23 (41.8) | 0.054 |
| Cognitive impairment | 90 (58.8) | 37 (57.8) | 34 (63.0) | 0.830 |
| Abnormal nutrition | 27 (17.6) | 13 (20.3) | 8 (14.5) | 0.729 |
| Baseline osteoporosis | 21 (14.3) | 11 (17.5) | 8 (14.8) | 0.800 |
| Fracture history | 5 (3.3) | 4 (6.2) | 2 (3.6) | 0.590 |
| Duration on ART > 20 year | 13 (8.5) | 7 (10.9) | 8 (14.5) | 0.385 |
| ARV regimen |  |  |  | 0.187 |
| NNRTI-based | 86 (56.2) | 41 (64.1) | 26 (47.3) |  |
| PI-based/ INSTI-based/ Others | 67 (43.8) | 23 (35.9) | 29 (52.7) |  |
| Current CD4 <500 cell/mm^3^ | 53 (34.6) | 17 (26.6) | 6 (10.9) | 0.002 |
| Baseline IL-6 (pg/ml) | 5.8 (4.1-7.5) | 5.9 (2.8-8.0) | 5.9 (4.6-8.0) | 0.312 |
| Baseline high sensitivity CRP > 2, (mg/dl) | 45 (29.4) | 22 (34.4) | 24 (43.6) | 0.163 |
| Vitamin D deficiency (<20) (vs. >20) | 48 (31.8) | 24 (38.7) | 15 (27.3) | 0.412 |

**Note:** Categorical data are presented as frequency (percentage) and continuous data are presented as median (interquartile range)

**Abbreviations**: ASCVD: atherosclerotic cardiovascular disease; ART: antiretroviral therapy; NNRTI: non-nucleoside reverse transcriptase inhibitor; INSTI: integrase stand transfer inhibitors; PI: protease inhibitor; CRP: C-reactive protein; hs-CRP: high sensitivity CRP; IL-6: Interleukin-6

**Table S13: Factor associated with worsening and improvement frailty stage by using multinomial logistic regression in older adults living with HIV who** **completed the 5-year follow-up**

|  | **Outcome** | | **Outcome** | |
| --- | --- | --- | --- | --- |
|  | **worsening vs. stable** | | **improvement vs. stable** | |
|  | **OR (95%CI)** | **aOR (95%CI)** | **OR (95%CI)** | **aOR (95%CI)** |
| Age≥ 55 year | 1.15 (0.64-2.07) | 1.15 (0.63-2.13) | 1.11 (0.6-2.06) | 1.34 (0.69-2.60) |
| Female (vs. male) | 0.94 (0.52-1.73) | 0.85 (0.46-1.59) | 1.75 (0.94-3.25) | 1.49 (0.78-2.85) |
| Waist-hip ratio> 1 (vs <1) | 1.84 (0.70-4.83) |  | 0.49 (0.10-2.27) |  |
| Ever drink alcohol (vs. Not drink) | 1.05 (0.5-2.16) |  | 0.70 (0.30-1.63) |  |
| Ever Smoking (vs. never) | 1.19 (0.64-2.2) |  | 0.72 (0.36-1.45) |  |
| Hypertension | 0.93 (0.51-1.68) |  | 0.61 (0.31-1.17) |  |
| Diabetes mellitus | 1.25 (0.59-2.61) |  | 0.18 (0.04-0.80) |  |
| Multimorbidity | 1.13 (0.62-2.09) | 1.03 (0.54-1.94) | 0.46 (0.25-0.86) | **0.45 (0.23-0.87)** |
| Abnormal nutrition (vs. normal) | 1.19 (0.57-2.49) |  | 0.79 (0.34-1.87) |  |
| Duration on ART > 20 year | 1.32 (0.5-3.49) |  | 1.83 (0.72-4.7) |  |
| Current CD4 <500 cell/mm^3^ | 0.68 (0.36-1.30) | 0.64 (0.33-1.25) | 0.23 (0.09-0.57) | **0.24 (0.10-0.61)** |
| Baseline IL-6 (pg/ml) per a unit increase | 0.98 (0.93-1.02) |  | 1.00 (0.98-1.01) |  |
| Baseline hs-CRP > 2, (mg/dl) | 1.26 (0.67-2.34) |  | 1.86 (0.98-3.51) |  |
| Vitamin D deficiency (<20) (vs. >20) | 1.36 (0.73-2.51) | 1.39 (0.75-2.6) | 0.80 (0.41-1.6) | 0.92 (0.45-1.88) |

**Note:** Factors associated with worsening and improvement in frailty stage were examined using multinomial logistic regression. Multimorbidity was used instead of individual comorbidities to avoid multicollinearity.

**Abbreviations**: OR: odds ratio; Adj OR: adjusted odds ratio; CI: confidence interval; ART: antiretroviral therapy; IL-6: Interleukin-6; CRP: C-reactive protein; hs-CRP: high sensitivity CRP

**Figure S1:** **Prevalence of frailty at baseline and after 5 years of follow-up in older adults living with HIV who** **completed the 5-year follow-up**

**
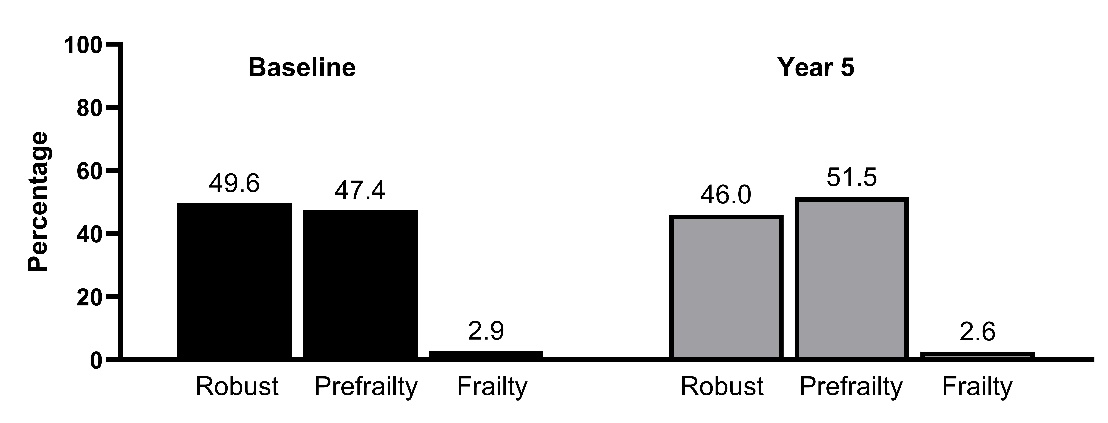
**

**Figure S2: Frailty status at baseline and the transition to year 5 in older adults living with HIV who** **completed the 5-year follow-up**


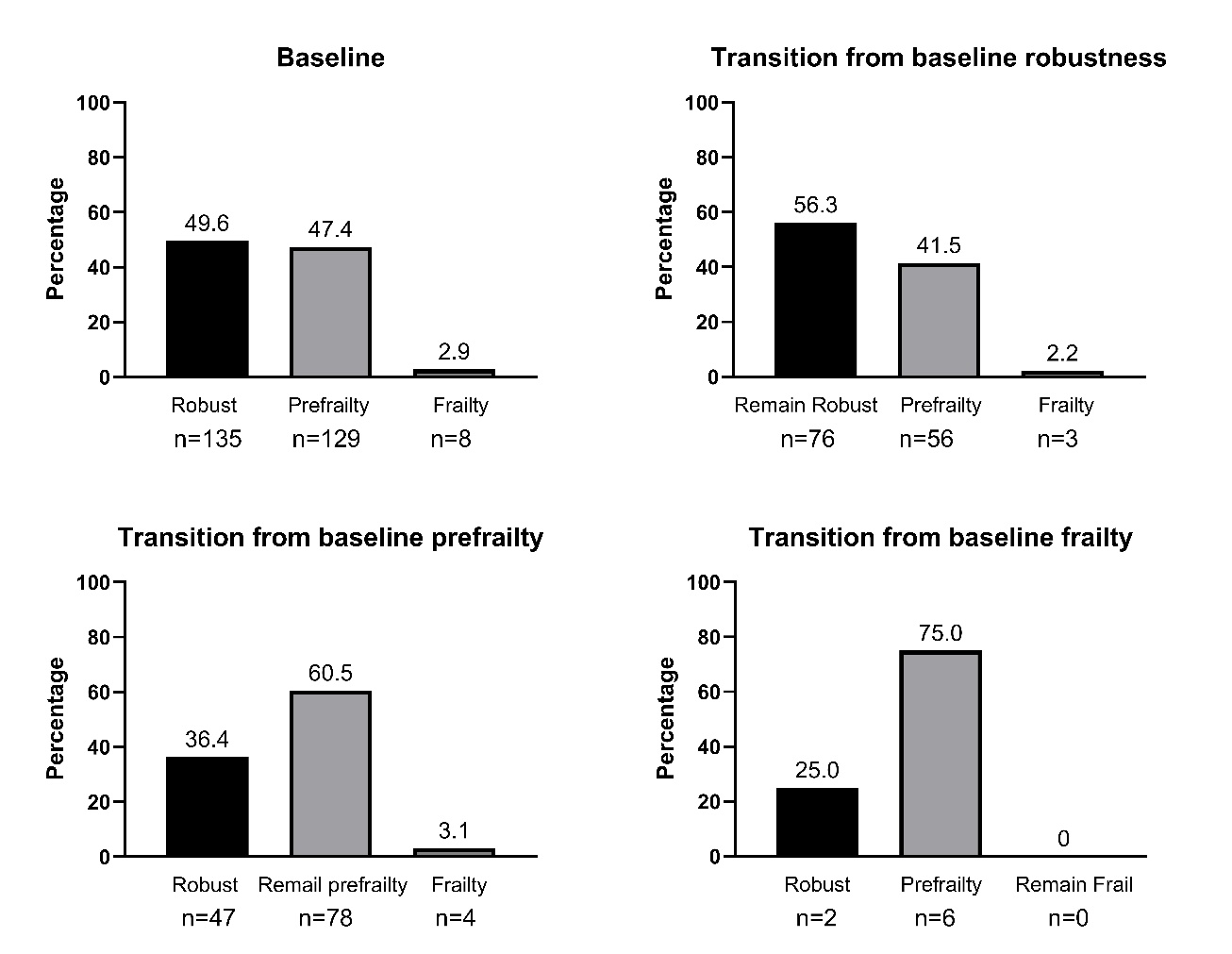

Supplement: Supplementary file 1 — Table S1: Baseline characteristics of PWH with and without frailty assessment at follow‐up visit Table S2: Demographic characteristics by frailty status at 5‐year follow‐up Table S3: Factors associated with frailty by using generalized estimating equation in older adults living with HIV Table S4: Factors associated with prefrailty to frailty using generalized estimating equation Table S5: Demographics and clinical factors by frailty transition state from baseline to 5‐year follow‐up Table S6: Factors associated with worsening and improvement of the frailty stage using multinomial logistic regression Table S7: Factors associated with worsening stage among participants with baseline robust/pre‐frail (n = 311) using logistic regression Table S8: Factors associated with reversal stage among participants with baseline pre‐frail/frail (n = 166) using logistic regression Table S9: Demographic characteristics by frailty status at 5‐year follow‐up in older adults living with HIV who completed the 5‐year follow‐up Table S10: Factors associated with frailty using generalized estimating equation (GEE) in older adults living with HIV who completed the 5‐year follow‐up Table S11: Factors associated with prefrailty to frailty using generalized estimating equation (GEE) in older adults living with HIV who completed the 5‐year follow‐up Table S12: Associations of demographics and clinical factors by frailty transition state from baseline to 5‐year follow‐up in older adults living with HIV who completed the 5‐year follow‐up Table S13: Factors associated with worsening and improvement of the frailty stage using multinomial logistic regression in older adults living with HIV who completed the 5‐year follow‐up Figure S1: Prevalence of frailty at baseline and after 5 years of follow‐up in older adults living with HIV who completed the 5‐year follow‐up Figure S2: Frailty status at baseline and the transition at 5 years of follow‐up among older adults living with HIV who completed the 5‐ye [file JIA2-29-e70099-s001.docx]
